# Comparative outcomes of minimally invasive right anterior mini-thoracotomy vs conventional sternotomy in aortic valve replacement: a propensity matched meta-analysis

**DOI:** 10.1097/MS9.0000000000003870

**Published:** 2025-09-15

**Authors:** Barka Sajid, Rabeya Farid, Muhammad Affan, Ali Abdullah, Dua Zehra, Kashmala Rahl, Zainab Wahaj, Aliza Asad, Ahzam Khan Ghori, Hafsa Jawaid, Khabab Abbasher Hussien Mohamed Ahmed

**Affiliations:** a Jinnah Sindh Medical University, Karachi, Pakistan; b Faculty of Medicine, University of Khartoum, Khartoum, Sudan

**Keywords:** aortic valve replacement, conventional sternotomy, minimally invasive right anterior mini-thoracotomy, propensity matched studies, surgical outcomes

## Abstract

**Background:**

Aortic valve replacement (AVR) is the standard intervention for treating aortic valve pathologies like aortic stenosis, with conventional sternotomy (CS) being the standard approach. Despite its efficacy, it is associated with post-procedural complications. Hence, a novel minimally invasive procedure called right anterior mini-thoracotomy (RAMT) has emerged, minimizing surgical trauma and enhancing recovery. The emergence of RAMT offers new dimensions to surgical decision-making.

**Methods:**

Electronic databases such as PubMed, Cochrane Library, and ScienceDirect were searched from inception to June 2024 for propensity matched studies comparing RAMT with CS for AVR. The Newcastle–Ottawa Scale was used to assess the quality of included studies and to assess the certainty of the outcomes measured, GRADE assessment was performed. Statistical analysis was performed using RevMan (version 5.4.1), and risk ratio (RR) and weighted mean difference (WMD) with 95% CIs were utilized using the random effects model.

**Results:**

Five propensity matched retrospective studies encompassing 1691 patients were included. RAMT exhibited superiority in terms of mortality (RR: 0.51; 95% CI: 0.26 to 0.99; *P* = 0.05) and ventilation time (WMD: −1.19, 95% CI: −2.23 to −0.14; *P* = 0.03). Conversely, outcomes such as hospital stay, reexploration for bleeding, aortic clamping time, ICU length of stay, atrial fibrillation, infection, stroke, and bypass time demonstrated no statistically significant differences between RAMT and CS.

**Conclusions:**

Our study found RAMT a suitable alternative as it effectively reduced early mortality and ventilation time; however, high heterogeneity among the studies and limited data suggest that further research is warranted to confirm its efficacy.

## Introduction

Aortic stenosis is characterized by aortic valve constriction and occlusion of blood flow leading to significant cardiac dysfunction extending beyond the left ventricle^[1]^. Aortic stenosis is currently one of the most common and potentially fatal valvular heart disorders due to aging populations and longer life expectancies. Since symptomatic chronic disease is always deadly if not treated promptly, early detection and therapy of aortic stenosis are crucial. Currently, there is a dearth of noninvasive treatments; in fact, symptomatic pharmacological treatment has failed to show evidence that it may halt aortic stenosis; only surgical alternatives, such as aortic valve replacement (AVR) therapy, are available, which entails considerable postoperative problems^[2]^.

**HIGHLIGHTS**

- Aortic stenosis is a potentially fatal valvular disorder driven by increased life expectancy.
- The prevalent approach for aortic valve replacement (AVR) is conventional aortic valve replacement (CAVR).
- The novel right anterior mini-thoracotomy (RAMT) reduces surgical trauma and enhances recovery.
- RAMT, a minimally invasive surgical procedure, has introduced new dimensions to surgical decision-making.

Although there have been significant advancements in interventional treatment options recently, the standard surgical procedure for AVR is conventional aortic valve replacement (CAVR), which involves a conventional (full) sternotomy (CS)^[3]^. The most prevalent surgical method for AVR is CAVR, which involves performing a full sternotomy through an incisional exposure of the sternum and replacing the valve while on cardiopulmonary bypass (CPB). The median CS is well tolerated; however, as less invasive options become available, smaller surgical incisions are considered^[4]^. Additionally, full sternotomy provides a high risk for complications during recovery^[3]^.

Over the past few decades, minimally invasive surgeries have gained immense popularity, especially right anterior mini-thoracotomy (RAMT). These procedures preserve the integrity of the sternum, and also minimize postoperative complications especially atrial fibrillation (AF)^[5]^. They also shorten hospital stays and ventilation times, lower mortality rate, require fewer blood transfusions, produce better cosmetic results, and speed up recovery times^[6]^. However, they also had certain potential risks, like longer CPB and cross clamp periods, and some patients had also reported groin complications as a result of prolonged peripheral cannulation^[7]^. Furthermore, RAMT may propose significant risks to patients with chest wall abnormalities and prior lung diseases as the patient cannot survive on single lung ventilation. When requiring additional operations such as coronary artery bypass grafting or multiple valve operations or in the presence of peripheral artery disease, standard approach is often more beneficial^[8]^.

Previously, meta-analyses comparing CS with minimally invasive surgeries have been conducted, providing efficacy of minimally invasive surgeries. However, due to low sample size and insufficient data, there is no comprehensive research article that solely and statistically demonstrates the potential of RAMT despite showing substantial benefits over CS in terms of postoperative outcomes^[9]^. Due to global intensification of this devastating disease, medical community is keen to find out most appropriate and yet minimally invasive surgeries that can treat aortic stenosis with reduced complications. Therefore, this meta-analysis aims to compare perioperative outcomes by incorporating propensity matched retrospective cohorts for patients undergoing AVR by CS or minimally invasive RAMT^[10]^.

## Methods

### Data sources and search strategy

This systematic review and meta-analysis were conducted in accordance with the Preferred Reporting Item Review and Meta-Analysis (PRISMA) guidelines^[11]^. This study is registered in the PROSPERO database with registration ID: CRD4202457442. Literature search was conducted across various electronic databases including PubMed, Cochrane Library and Science Direct from inception to June 2024. These databases were searched using both medical subject headings (MeSH) and entry terms that included: [aortic valve”(MeSH) OR “aortic”] AND [“replace” OR “replaced” OR “replacement” OR “replantation”(MeSH) OR “AVR”) AND “right” AND “anterior” AND “mini” AND [“thoracotomy”(MeSH) OR “thoracotomies” OR “RAMT”] AND “conventional” AND [“sternotomy”(MeSH) OR “sternotomies” OR “full” AND “sternotomy”(MeSH). The detailed search strategies for each database are mentioned in the Supplemental Digital Content Table 1, Available at, http://links.lww.com/MS9/A941.

### Study screening and eligibility criteria

Studies with the relevant keywords were transferred to Rayyan AI for further screening. Only articles available in the English language were considered, without any restrictions on the date of publication. Duplicate articles were extracted and eliminated. Additionally, bibliographies of the included studies were cross-checked to ensure no relevant articles were overlooked. Two independent investigators (A.G and A.A) performed screening, starting at title and abstract level, followed by the full-text assessment. The eligibility criteria were established through consensus between the two primary authors, with an additional author (B.S.) resolving any discrepancies. Included articles focused on patients above the age of 18 and who underwent AVR either by CS (full sternotomy) or RAMT. The exclusion criteria consisted of patients requiring concomitant procedures beyond AVR, patients who underwent transcatheter aortic valve implantation and participants with previous cardiac or thoracic surgery. For this meta-analysis, studies such as randomized controlled trials (RCTs), observational studies, case–control studies and cohorts were considered with exclusion of editorials, systematic reviews, letter to editors, and case reports.

Additionally, approval from the ethical committee was not a requirement for our systematic review and meta-analysis.

### Data extraction and risk of bias assessment

Two authors (R.F and B.S) independently harvested relevant data on an extraction sheet constructed on Google sheets with disparities being resolved by an additional author (A.I). The variables consisted of name of the author, study type, location, publication year and patient demographics such as, gender, age, body mass index (BMI), and associated comorbidities. Moreover, outcomes – for instance, ICU length of stay, hospital stay, mortality, and complications such as bleeding, stroke, infection, AF, and transfusion details – were recorded, along with procedural times like aortic cross-clamping (ACC) and bypass duration. To evaluate the quality of the retrospective cohort studies, the Newcastle–Ottawa Scale (NOS) was utilized^[12]^. The bias tool reviewed studies on the basis of selection, comparability, and outcomes with a star grading system of 7 or above indicating a high quality. To assess the certainty of the outcomes measured, GRADE (Grading of Recommendations Assessment, Development and Evaluation) assessment was performed using GRADEpro GDT^[13]^.

### Statistical analysis

Clinical outcomes were analyzed using the latest version of Review Manager software (version 5.4.1), employing risk ratio (RR) and weighted mean difference (WMD) for dichotomous and continuous outcomes respectively, with 95% confidence intervals (CI). Random effects model was used to address variability among the included studies. A *P*-value of ≤0.05 was considered as statically significant. Heterogeneity among the studies was assessed using the *I*^2^ Higgins Test, where studies with *I*^2^ >50% were deemed heterogeneous warranting further investigation through the sensitivity analysis using leave-one-out method^[14]^. In cases of substantial heterogeneity, potential clinical and methodological factors were qualitatively explored.

## Results

### Search results

**Figure 1.**
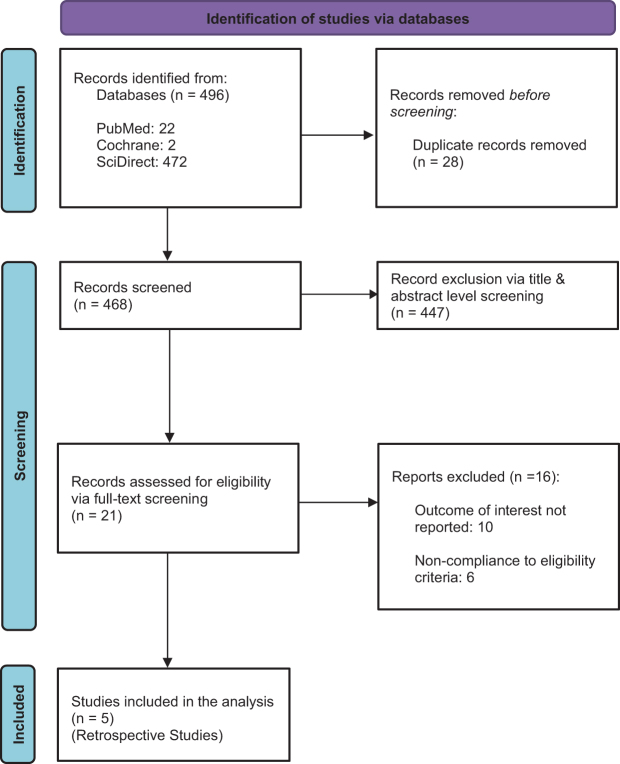
PRISMA flowchart illustrating the study screening process.

A total of 496 references were identified through three electronic database searches. After screening for duplicates, 468 studies remained. A total of 447 studies were excluded on the basis of title and abstract. All of the remaining articles were screened in full text. Finally, five studies were deemed eligible for this meta-analysis^[3,5,9,15,16]^. The PRISMA flowchart summarizes our study selection in Fig. 1.

### Study characteristics

Five studies, comprising a total of 1691 patients, were pooled in this meta-analysis. All studies were retrospective studies. Among these, 921 (54.4%) patients were in the RAMT group, while 770 (45.5%) were in the CS group. Follow-up period was mentioned only in Glauber *et al*, which was 30 months. Following comparisons were made between RAMT and CS: aortic valve stenosis, left ventricular ejection fraction %, New York Health Association Functional Classification (NYHA), chronic obstructive pulmonary disease (COPD), diabetes mellitus, dyslipidemia, hypertension, BMI, gender, and age (Table 1).

**Table 1:** Baseline Characteristics of the Included Studies.

Author	Publication year	Study design	Study duration	Location	Patient sample			Age in years (SD/IQR)		Male %		Female %		BMI in kg/m^2^ (SD)		Hypertension, *n* (%)		Dyslipidemia, *n* (%)		Diabetes Mellitus, *n* (%)		COPD, *n* (%)		NYHA Class 3-4, *n* (%)		Aortic valve stenosis, *n* (%)	
					Total	CS	MIAVR	CS	MIAVR	CS	MIAVR	CS	MIAVR	CS	MIAVR	CS	MIAVR	CS	MIAVR	CS	MIAVR	CS	MIAVR	CS	MIAVR	CS	MIAVR
Gilamanov *et al*^[9]^	2015	Retrospective cohort	Feb 2001 till Sept 2013	Italy	200	100	100	82.5(2.2)	83 (2.1)	33	36	67	64	25.6 (3.8)	25.6 (3.5)	85(85)	76(76)	53(53)	61(61)	12(12)	17(17)	15(15)	11(11)	48(48)	39(39)	84(84)	89(89)
Seitz *et al*^[5]^	2017	Retrospective cohort	Jan 2013 till Oct 2016	Australia	106	53	53	72 (11.6)	73 (8.8)	47.16	50.94	52.84	49.06	27.4 (4.7)	28 (5.6)	27(50.9)	35(66)	N/A	N/A	9(17)	10(18.9)	N/A	N/A	15(28.3)	16(30.2)	N/A	N/A
Olds *et al*^[3]^	2019	Retrospective cohort	Jan 2012 till Dec 2015	USA	383	116	267	74 (62.5-80)	75 (67-81)	58.6	57.3	41.4	42.7	N/A	N/A	95(81.9)	240(89.9)	94(81)	214(80.1)	43(37.1)	77(28.8)	N/A	N/A	N/A	N/A	101(87.1)	246(92.1)
Glauber *et al*^[15]^	2013	Retrospective cohort	January 2005 to July 2010	Italy	276	138	138	69.8 (11.6)	69.5 (12.4)	60.9	57.9	39.1	42.1	1.87 (0.2)	1.85 (0.2)	110(79.7)	110(79.7)	N/A	N/A	33(23.9)	27(16.9)	22(15.9)	22(15.9)	46(33.3)	41(30)	64(46.3)	61(44.2)
Giglio *et al*^[16]^	2018	Retrospective cohort	January 2010 and May 2016	Italy	726	363	363	72.7 (9.8)	73.4 (10.3)	48.2	54.5	51.8	45.5	27.1 (4.3)	27.2 (4.5)	260(71.6)	267(73.6)	192(52.9)	183(50.4)	74(20.4)	75(20.7)	31(8.5)	35(9.6)	N/A	N/A	N/A	N/A

### Risk of bias and GRADE assessment

The NOS was used to assess the studies included as shown in Supplemental Digital Content Table 2, Available at, http://links.lww.com/MS9/A941^[12]^. It primarily serves as a tool to assess the quality, having a star system (range, 0–9). NOS has three parameters to assess the quality: selection, comparability, and exposure/outcome assessment. A study is said to be of high quality if it has a score of 7 or above, and a score of 6 or below indicates a low-quality study. All the five studies included in meta-analysis were high quality studies. To determine the certainty of the included evidence, GRADE assessment was also performed^[13]^. Outcomes such as ventilation time, ACC time, and mortality depict a very low certainty of evidence, whereas CPB time, AF, infection, stroke, reexploration for bleeding, hospital stay, and ICU length of stay show evidence of low certainty due to high heterogeneity and wide CI. The summarized table for the GRADE assessment is provided in Supplemental Digital Content Table 3, Available at, http://links.lww.com/MS9/A941.

### Outcomes

After a comprehensive review, five papers that met our inclusion criteria were selected. We analyzed 10 outcomes among RAMT and CS. The primary outcomes include mortality, hospital stay, and reexploration for bleeding, whereas the secondary outcomes include ACC time, ventilation time (hours), ICU length of stay, AF, infection, stroke, and bypass time.

#### Mortality

**Figure 2.**
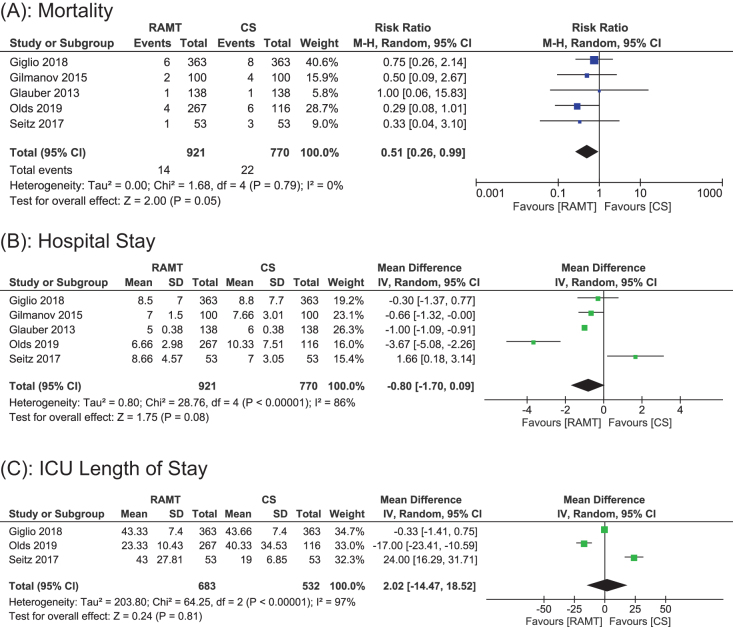
Forest plot displaying the outcome for (a) mortality, (b) hospital stay, and (c) ICU stay.

Data for early mortality in hospital were reported in all five studies, including a total of 1691 patients (921 RAMT vs 770 CS). A random effects model was used for analysis, representing a statistically significant result, showing considerably lower early mortality after RAMT than CS (RR: 0.51; 95% CI: 0.26 to 0.99; *P* = 0.05; *I*^2^ = 0%; Fig. 2A). The results did not demonstrate significant heterogeneity.

#### Hospital stay

Data for overall length of stay in the hospital were reported in all five studies, including a total of 1691 patients (921 RAMT vs 770 CS). Our pooled analysis showed statistically nonsignificant reduction in hospital stay in RAMT as compared to CS (WMD: −0.80; 95% CI: −1.70 to 0.09; *P* = 0.08; Fig. 2B). The results showed significant heterogeneity (*I*^2^ = 86%). A sensitivity analysis was carried out to identify the potential source of heterogeneity, but removal of individual studies did not significantly reduce the *I*^2^ value. This indicates that the heterogeneity was not caused by a single study; instead it may have arisen from multiple study level differences such as variations in health care settings, discharge practices of institutions or patient characteristics.

#### ICU length of stay (hours)

Data for ICU length of stay were reported in three studies, including a total of 1215 patients (683 RAMT vs 532 CS). A random effects model was used for pool analysis that showed no statistically significant difference in ICU stay between the RAMT and CS groups (WMD: 2.02 h; 95% CI: −14.47 to 18.52; *P* = 0.81; Fig. 2C). Additionally, significant heterogeneity (*I*^2^ = 97%) was reported among studies, indicating high variability among the studies. Due to heterogeneity caused by more than one study, sensitivity analysis did not significantly change the results. This indicates that this high heterogeneity was due to inter study differences such as diverse patient characteristics and perioperative management strategies, etc.

#### CPB time (minutes)

**Figure 3.**
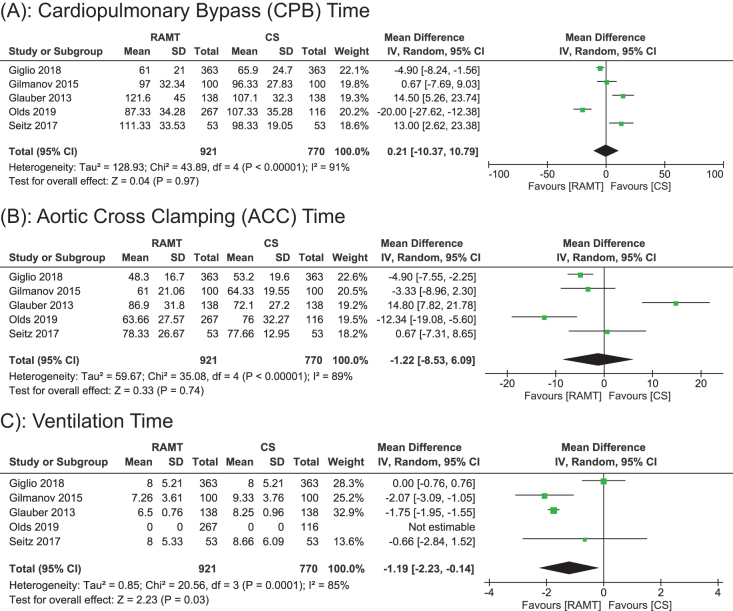
Forest plot displaying the outcome for (a) cardiopulmonary bypass (CPB) time, (b) aortic cross-clamping (ACC) time, and (c) ventilation time.

Data for bypass time were included in all five studies, including a total of 1691 patients (921 RAMT vs 770 CS). A random effects model was used for analysis that indicated that RAMT did not significantly reduce bypass time compared to CS (WMD: 0.21 min; 95% CI: −10.37 to 10.79; *P* = 0.97; Fig. 3A). The heterogeneity was high (*I*^2^ = 91%), suggesting considerate variability among the studies. The sensitivity analysis did not support the robustness of the results, since multiple studies were the source of heterogeneity. This suggests that factors like differences in surgeon’s operative techniques, or institutional protocols may have influenced the outcome and limited the generalizability of pooled results.

#### ACC time

Data for aortic clamping time were reported in all 5 studies, including a total of 1691 patients, (921 RAMT vs 770 CS). Our pooled analysis showed a nonsignificant reduction in aortic clamping time in RAMT as compared to CS (WMD: −1.22; 95% CI: −8.53 to 6.09; *P* = 0.74; Fig. 3B). The results showed significant heterogeneity (*I*^2^ = 89%).

A sensitivity analysis (leave-out method) was performed on account of substantial heterogeneity. Glauber *et al* was excluded, decreasing the heterogeneity significantly (*I*^2^ = 56%) as shown in Supplemental Digital Content Fig. 1, Available at, http://links.lww.com/MS9/A941^[15]^. Furthermore, the overall pooled mean difference for ACC time was found to be (WMD: −5.10; 95% CI: −9.16 to −1.05; *P* = 0.01), favoring RAMT.

#### Ventilation time (hours)

Data for ventilation time was reported in all five studies, including a total of 1691 patients (921 RAMT vs 770 CS). Our pooled analysis showed a significant decrease in ventilation time in RAMT as compared to CS (WMD: −1.19, 95% CI: −2.23 to −0.14; *P* = 0.03; Fig. 3C]. However, the results demonstrated significant heterogeneity (*I*^2^ = 85%) among the studies.

A sensitivity analysis (leave-out analysis) was performed on account of substantial heterogeneity. Excluding the study by Giglio *et al* notably reduced the heterogeneity (*I*^2^ = 0) as shown in Supplemental Digital Content Figure 2, Available at, http://links.lww.com/MS9/A941^[16]^. Furthermore, the overall pooled mean difference for ventilation time showed a shorter duration in RAMT as compared to CS (WMD: −1.75; 95% CI: −1.95 to −1.55; *P* < 0.00001).

#### Reexploration of bleeding

**Figure 4.**
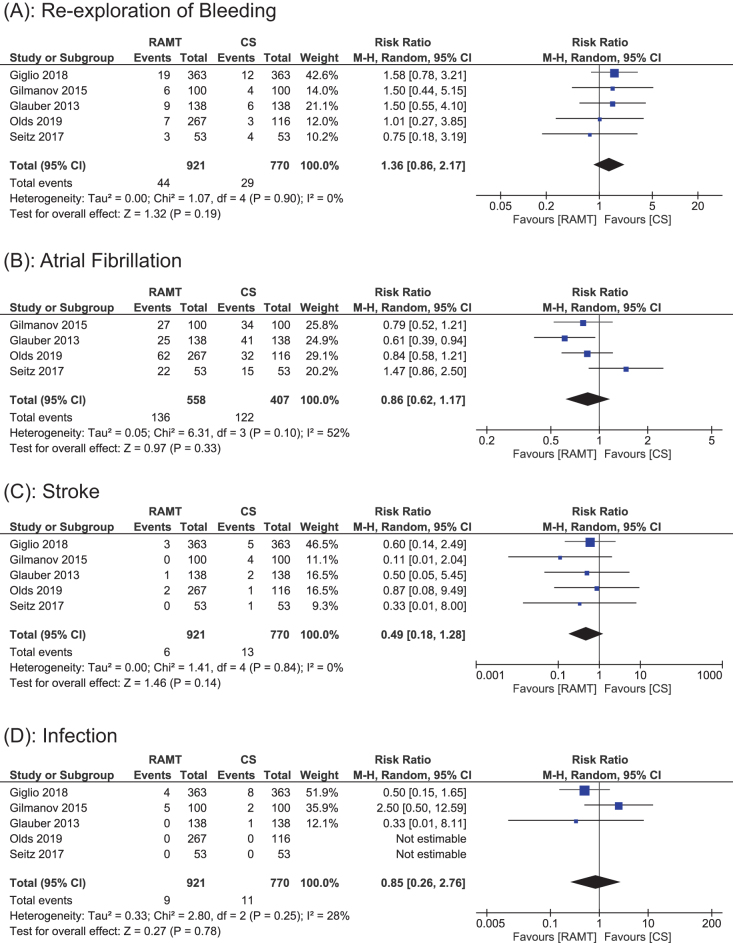
Forest plot displaying the outcome for (a) reexploration for bleeding, (b) atrial fibrillation, (c) stroke, and (d) infection.

Data for reexploration for bleeding were reported in all five studies, including a total of 1691 patients (921 RAMT vs 770 CS). RAMT increased the incidence of reexploration for bleeding, which is not significant when compared to CS (RR: 1.36; 95% CI: 0.86 to 2.17; *P* = 0.19; *I*^2^ = 0%; Fig. 4A). The results did not demonstrate significant heterogeneity.

#### Atrial fibrillation

Data for AF were included in four studies, including a total of 965 patients (558 RAMT vs 407 CS). A random effects model was used for pooled analysis that showed a reduction in the incidence of AF with RAMT; however, this association was found to be insignificant (RR: 0.86; 95% CI: 0.62 to 1.17; *P* = 0.33; Fig. 4B). Additionally, moderate heterogeneity (*I*^2^ = 52%) was reported, indicating some variability among studies.

#### Stroke

Data for stroke rates were reported in all five studies, including a total of 1691 patients (921 RAMT vs 770 CS). A random effects model was used for the pooled analysis, showing no statistically significant difference in stroke rates between the RAMT and CS groups (RR: 0.49; 95% CI: 0.18 to 1.28; *P* = 0.14; Fig. 4C). The analysis reported no heterogeneity among the studies (*I*^2^ = 0%).

#### Infection

Data for incidence of infection were reported in three studies, including a total of 1691 patients (921 RAMT vs 770 CS). A random effects model was used for analysis, showing an insignificant difference in infection rates between the RAMT and CS groups (RR: 0.85; 95% CI: 0.26 to 2.76; *Z* = 0.27; *P* = 0.78; Fig. 4D). Additionally, moderate heterogeneity (*I*^2^ = 28%) was reported among the studies, indicating some variability in the studies.

## Discussion

Our meta-analysis provides a comparative evaluation of RAMT versus CS for AVR. Five studies, in total, were analyzed, allowing us to draw several observations in regard to the efficacy and safety of RAMT compared with CS. Minimally invasive aortic valve surgery has generally been accepted by the surgical community, with patients frequently expecting it to be offered as an alternative to CS. This is largely based on small prospective and single-center retrospective trials comparing AVR via CS and with minimally invasive approaches^[17,18]^. Several meta-analyses also have been conducted on the topic, focusing on various approaches for AVR, with the most recent one being carried out in 2024^[10,19,20]^. The first AVR via right thoracotomy was reported, in 1993, by Rao and Kumar^[21]^. The technique which uses a small anterior thoracotomy in the third intercostal space was described, in 1997, by Benetti^[22]^. RAMT might be preferred for various reasons including cosmesis, ability to centrally cannulate, ease of surgical exposure and the perception that it is associated with favorable outcomes and patient satisfaction^[6]^.

Our analysis found a significantly lower early mortality rate for RAMT when compared with CS. This finding is of clinical importance as this suggests that RAMT offers a survival advantage in the perioperative period. The complete lack of heterogeneity also reinforces the reliability of this outcome across different studies. The decrease in mortality might be due to the less invasive nature of RAMT, which may minimize surgical trauma and associated complications^[23]^. This finding also aligns with previous research which shows that less invasive procedures may reduce mortality^[6]^. Early postoperative mortality is the most crucial determinant of the success of the procedure and patient prognosis following AVT. A nearly 50% reduction in mortality following RAMT as shown in our study, highlights that less invasive procedures may reduce surgical trauma, postoperative bleeding, systemic inflammation and infection, thus reducing the risk of early mortality^[24]^. It is important to mention that although RAMT may alter immediate results, other factors such as age, AF, left ventricular dysfunction, and prior cardiovascular illness may have an effect on long-term survival, as indicated in previous literature^[25,26]^. Some studies have revealed a nonsignificant relationship between early mortality and RAMT, e.g., Khalid *et al*, while Phan *et al* found an almost comparable relationship^[18,19]^.

Even though the difference was not statistically significant, a trend toward a shorter hospital stay for patients undergoing RAMT was seen. The significant heterogeneity, however, suggests significant variability across the studies. This lack of consistency in findings can be attributed to differences in perioperative care, patient selection, and institutional protocols. Previous studies have shown significantly shorter hospital stays for patients undergoing RAMT^[6,10,19,20,27]^. This should be explored in future studies, with standardized protocols, to better understand its impact on hospital stay.

Reexploration for bleeding was found to be more frequent in the RAMT group, but the increase was not statistically significant. Heterogeneity was completely absent, indicating consistency across studies. The increased risk, even if not significant, warrants consideration as it could reflect the technical challenge associated with the minimally invasive approach^[28,29]^. Possible reasons for the increase in bleeding may include injury to the right internal mammary artery, injury to intercostal vessels and increased aortic clamp time^[20]^. An observational study consisting of 566 propensity matched pairs undergoing heart surgery found that reoperation caused a significantly increased postoperative mortality, complications, reoperations for valve dysfunctions, and longer hospital stays^[30]^. The surgeon’s experience with such techniques could play a role in minimizing this risk^[31]^. The meta-analysis by Salmasi *et al* was the first to draw attention to an increased risk associated with RAMT, which is in line with another study by Ogami *et al*^[20,32]^.

After sensitivity analysis, by excluding Glauber *et al*, a statistically significant reduction in aortic clamping time favoring RAMT was revealed. This suggests that RAMT offers more efficient clamping times and also indicates a more consistent benefit of RAMT in reducing aortic clamping times across the remaining studies. However, excluding this study suggests potential variations in surgical techniques, clinical practices, or patient selection criteria which may influence the generalizability of these results to the healthcare settings. Nevertheless, potential reasons for this can be improved surgical techniques in less invasive approaches. This is in contrast to previous studies which showed an increased aortic clamping time^[10,33]^. The study by Khalid *et al* showed no significant relationship between aortic clamping time and RAMT^[19]^. In addition, RAMT significantly reduced ventilation time compared to CS, with heterogeneity down to zero, after removal of Giglio *et al*. This is important as it suggests RAMT facilitates faster recovery of respiratory function and reduced risk of complications associated with ventilator care including pneumonia and atelectasis^[27]^. Reasons for this can be reduced surgical trauma and decreased pain, which aligns with the known benefits of minimally invasive surgery^[23]^. Even so, the exclusion of the study by Giglio *et al* underscores potential differences in perioperative care and ventilation protocols across different healthcare centers, suggesting that these findings should be applied cautiously and in the context of institution-specific practices. Our analysis showed no significant difference in the length of ICU stay between the two approaches, with considerable heterogeneity also being present. This indicates that the effect of surgical approach on the length of ICU stay is highly variable and may depend on several factors, e.g., comorbidities, postoperative complications as well as institutional practices.

Although RAMT was associated with a reduction in AF, the result was not statistically significant and had moderate heterogeneity. AF is a common complication that occurs after AVR and while RAMT might reduce it due to limited pericardial incision and reduced inflammation, the evidence in our analysis does not strongly support this^[10,34]^. The variability in our results can be due to the reasons already stated above. A significant decrease in AF due to RAMT has been demonstrated previously^[10]^. No statistically significant difference was also noted for infection rates between RAMT and CS, with moderate heterogeneity. It has been stated previously that minimally invasive procedures such as RAMT may have an advantage over CS in reducing infection and complications from wounds as the sternum and ribs are not disrupted^[35]^. The meta-analysis by Khalid *et al* also did not find evidence to support this^[19]^. Further studies that focus on infection rates themselves can clarify this finding.

Stroke rates also did not differ significantly between RAMT and CS. This suggests that both approaches have a comparable stroke risk, a critical outcome for AVR^[36]^. This is important as it shows that RAMT does not further compromise neurological outcomes. This is reassuring as there has been concern that RAMT, which requires retrograde arterial perfusion via femoral artery, is associated with an increased risk of stroke^[37]^. This also aligns with the understanding that stroke risk is influenced by patient factors such as atherosclerosis and surgical technique itself, rather than invasiveness^[38]^. Previous research has shown that comparable stroke rates exist for conventional AVR and RAMT^[18]^. A significant difference in bypass time between the two approaches was also not noted, with a high heterogeneity. Such variability can be expressed by reasons already stated such as patient complexity and institutional protocols. The absence of a clear advantage means that while it may offer other benefits, bypass time is not necessarily reduced.

Our meta-analysis demonstrates significant benefits in perioperative outcomes of RAMT over CS, particularly reduced early mortality and shorter ventilator time. However, the impact of these procedures on long-term patient outcomes remains unclear. Most of the included studies reported only short-term in-hospital outcomes, with the exception of one study that reported outcomes beyond 30 months. Nevertheless, the observed reduction in surgical trauma, faster recovery, and fewer perioperative and immediate postoperative complications suggest potential improvements in long-term outcomes, although robust data from RCTs remain limited^[39]^. A study by Diana Reser *et al* reported survival rates of 95.8% and 79% at 1 and 7 years, respectively, in patients undergoing RAMT. Freedom from major adverse cardiac and cerebrovascular events was 98.1% and 95.7%, while the reduced need for re-operation was 99.5% and 98.7%, respectively^[40]^. Similarly, a propensity-matched cohort demonstrated significantly higher 5- and 8-year survival rates in patients who underwent minimally invasive sternotomy^[41]^. Future studies incorporating long-term outcomes, such as survival, valve durability, and functional capacity, are necessary to better understand the comparative advantages of RAMT versus CS.

Our sensitivity analysis revealed several potential sources of heterogeneity across the included studies. Variations in patient selection criteria including differences in comorbidities, age distribution, and disease severity may have influenced outcomes. Similarly, surgical expertise and institutional protocols could have impacted operative times and complication rates. These differences make it challenging to draw firm conclusions from pooled retrospective data. Therefore, there is a pressing need for well-designed RCTs using standardized methodologies to reduce variability and provide more reliable comparisons.

The results of this meta-analysis have clinical and practical significance which can influence decision making for guiding surgical approaches for AVR. The benefit of RAMT over CS in reducing mortality rate and ventilation time suggests that this technique may have better perioperative outcomes by improving patient recovery and reducing the risk of complications associated with prolonged ventilator use. However, outcomes such as ICU stay, hospital stay, and reexploration for bleeding did not differ significantly, with considerable variability between studies. This indicates that the potential benefits of RAMT are likely influenced by factors such as surgeon experience, patient characteristics, and institutional practices. Therefore, the selection of RAMT and CS should be tailored to each patient’s profile and the availability of surgeon expertise and institutional practices.

### Strengths and limitations

There are several strengths in our study. First, it provides a comprehensive evaluation of multiple clinically relevant outcomes such as mortality, hospital stay, ventilation time, aortic clamping time, and various complications. Second, the pooled analysis included a total of 1691 patients. This is a relatively large number for a surgical meta-analysis, which enhances the statistical power and reliability, especially for outcomes with low event rates, such as mortality. Additionally, despite the retrospective nature, all included studies were assessed as high quality using the NOS.

Some limitations must be acknowledged. First, all five studies included were retrospective. This inherently limits the quality of evidence, as retrospective studies are prone to selection bias and the influence of confounding variables that may distort the true relationship between surgical protocols and outcomes. These confounding factors include variability in surgeon expertise across institutions and differences in institutional protocols, such as perioperative management and ICU care. Moreover, retrospective data rely on the accuracy of collected clinical information, which can also vary across institutions. These limitations in data accuracy and the inability to establish causality from observational studies underscore the need for RCTs comparing the efficacy of RAMT and CS.

Second, the analysis only included five studies, which limits the statistical power and generalizability of the results. Third, significant heterogeneity was observed in some outcomes such as hospital stay, aortic clamping time, ventilation time, ICU time, and bypass time. This shows that differences in study designs, patient populations, techniques used, and perioperative protocols may have influenced our findings. Fourth, only one study reported a follow-up period which limits the ability to assess long-term outcomes and complications. Finally, the absence of RCTs is a significant limitation, as they are the gold standard for comparing intervention, and reliance on observational studies limits the confidence in the findings.

## Conclusion

The number of RAMT procedure reports is increasing. Between 1998 and 2009, there were 34 trials, of which only 5 were of RAMT^[6]^. From 2010 to 2015, there were 18 reports, of which 12 were of RAMT^[6]^. As the prevalence of aortic stenosis in the elderly population is increasing, minimally invasive approaches are becoming more important^[42,43]^. Future research should focus on conducting RCTs to provide a higher quality of evidence on the effectiveness of RAMT versus CS. Perioperative care protocols and follow-up periods should be standardized in these trials to reduce variability and provide more accurate assessments of long-term outcomes. Furthermore, studies should focus on the impact of surgeon experience and institutional factors on the success of RAMT as these may be key determinants of patient outcomes. Research into outcomes reported by the patients themselves and quality of life following these procedures would also be valuable. Finally, investigating strategies to minimize the risk of reexploration for bleeding in RAMT could further enhance the safety profile of this technique.

## Data Availability

The data that support the findings of this manuscript are publicly available within the manuscript and the supplementary material.

## Research registration unique identifying number (UIN)

(1) Name of the registry: Not required (because this was a meta-analysis); (2) Unique Identifying number or registration ID: Not applicable (because this was a meta-analysis); (3) Hyperlink to your specific registration (must be publicly accessible and will be checked).

## References

[1] Fukui M Généreux P Cavalcante JL. Assessment of cardiac damage in aortic stenosis. Cardiol Clin 2020;38:23–31. 31753174 10.1016/j.ccl.2019.09.001

[2] Joseph J Naqvi SY Giri J. Aortic stenosis: pathophysiology, diagnosis, and therapy. Am J Med 2017;130:253–63. 27810479 10.1016/j.amjmed.2016.10.005

[3] Olds A Saadat S Azzolini A. Improved operative and recovery times with mini-thoracotomy aortic valve replacement. J Cardiothorac Surg 2019;14:91. 31072356 10.1186/s13019-019-0912-0 PMC6509756

[4] Kirmani BH Jones SG Malaisrie SC. Limited versus full sternotomy for aortic valve replacement. Cochrane Database Syst Rev 2017;4:CD011793. 28394022 10.1002/14651858.CD011793.pub2 PMC6478148

[5] Seitz M Goldblatt J Paul E. Minimally invasive aortic valve replacement via right anterior mini-thoracotomy: propensity matched initial experience. Heart Lung Circ 2019;28:320–26. 29291961 10.1016/j.hlc.2017.11.012

[6] Bowdish ME Hui DS Cleveland JD. A comparison of aortic valve replacement via an anterior right minithoracotomy with standard sternotomy: a propensity score analysis of 492 patients. Eur J Cardiothorac Surg 2016;49:456–63. 25750007 10.1093/ejcts/ezv038 PMC4711701

[7] Balmforth D Harky A Lall K. Is ministernotomy superior to right anterior minithoracotomy in minimally invasive aortic valve replacement? Interact Cardiovasc Thorac Surg 2017;25:818–21. 29049755 10.1093/icvts/ivx241

[8] Issa HMN, and Ruel M. Minimally invasive aortic valve replacement through a right anterior thoracotomy. Multimed Man Cardiothorac Surg 2024. doi:10.1510/mmcts.2024.041 38980286

[9] Gilmanov D Farneti PA Ferrarini M. Full sternotomy versus right anterior minithoracotomy for isolated aortic valve replacement in octogenarians: a propensity-matched study †. Interact Cardiovasc Thorac Surg 2015;20:732–41. 25757476 10.1093/icvts/ivv030

[10] Chang C Raza S Altarabsheh SE. Minimally invasive approaches to surgical aortic valve replacement: a meta-analysis. Ann Thorac Surg 2018;106:1881–89. 30189193 10.1016/j.athoracsur.2018.07.018

[11] Page MJ McKenzie JE Bossuyt PM. The PRISMA 2020 statement: an updated guideline for reporting systematic reviews. BMJ 2021;372:n71. 33782057 10.1136/bmj.n71 PMC8005924

[12] Stang A. Critical evaluation of the Newcastle-Ottawa scale for the assessment of the quality of nonrandomized studies in meta-analyses. Eur J Epidemiol 2010;25:603–05. 20652370 10.1007/s10654-010-9491-z

[13] GRADE handbook. cited [2024 Sep 24]. https://gdt.gradepro.org/app/handbook/handbook.html

[14] Higgins JPT Thompson SG. Quantifying heterogeneity in a meta-analysis. Stat Med 2002;21:1539–58. 12111919 10.1002/sim.1186

[15] Glauber M Miceli A Gilmanov D. Right anterior minithoracotomy versus conventional aortic valve replacement: a propensity score matched study. J Thorac Cardiovasc Surg 2013;145:1222–26. 22516391 10.1016/j.jtcvs.2012.03.064

[16] Del Giglio M Mikus E Nerla R. Right anterior mini-thoracotomy vs. conventional sternotomy for aortic valve replacement: a propensity-matched comparison. J Thorac Dis 2018;10:1588–95. 29707310 10.21037/jtd.2018.03.47 PMC5906311

[17] Mariscalco G Musumeci F. The minithoracotomy approach: a safe and effective alternative for heart valve surgery. Ann Thorac Surg 2014;97:356–64. 24263013 10.1016/j.athoracsur.2013.09.090

[18] Phan K Xie A Di Eusanio M. A meta-analysis of minimally invasive versus conventional sternotomy for aortic valve replacement. Ann Thorac Surg 2014;98:1499–511. 25064516 10.1016/j.athoracsur.2014.05.060

[19] Khalid S Hassan M Ali A. Minimally invasive approaches versus conventional sternotomy for aortic valve replacement in patients with aortic valve disease: a systematic review and meta-analysis of 17 269 patients. Ann Med Surg (Lond) 2024;86:4005–14. 38989160 10.1097/MS9.0000000000002204 PMC11230795

[20] Yousuf Salmasi M Hamilton H Rahman I. Mini-sternotomy vs right anterior thoracotomy for aortic valve replacement. J Card Surg 2020;35:1570–82. 32652784 10.1111/jocs.14607

[21] Rao PN Kumar AS. Aortic valve replacement through right thoracotomy. Tex Heart Inst J 1993;20:307–08. 8298332 PMC325118

[22] Benetti FJ Mariani MA Rizzardi JL. Minimally invasive aortic valve replacement. J Thorac Cardiovasc Surg 1997;113:806–07. 9104997 10.1016/S0022-5223(97)70246-0

[23] Murtuza B Pepper JR Stanbridge RD. Minimal access aortic valve replacement: is it worth it? Ann Thorac Surg 2008;85:1121–31. 18291224 10.1016/j.athoracsur.2007.09.038

[24] Ljungqvist O Scott M Fearon KC. Enhanced recovery after surgery: a review. JAMA Surg 2017;152:292–98. 28097305 10.1001/jamasurg.2016.4952

[25] Thourani VH Forcillo J Szeto WY. Outcomes in 937 intermediate-risk patients undergoing surgical aortic valve replacement in PARTNER-2A. Ann Thorac Surg 2018;105:1322–29. 29253463 10.1016/j.athoracsur.2017.10.062

[26] Rodriguez-Gabella T Voisine P Dagenais F. Long-term outcomes following surgical aortic bioprosthesis implantation. J Am Coll Cardiol 2018;71:1401–12. 29598859 10.1016/j.jacc.2018.01.059

[27] Lamelas J. Minimally invasive aortic valve replacement: the “Miami method. Ann Cardiothorac Surg 2015;4:71–77. 25694981 10.3978/j.issn.2225-319X.2014.12.10 PMC4311159

[28] Bonaros N Schachner T Lehr E. Five hundred cases of robotic totally endoscopic coronary artery bypass grafting: predictors of success and safety. Ann Thorac Surg 2013;95:803–12. 23312792 10.1016/j.athoracsur.2012.09.071

[29] Burt BM ElBardissi AW Huckman RS. Influence of experience and the surgical learning curve on long-term patient outcomes in cardiac surgery. J Thorac Cardiovasc Surg 2015;150:1061–7,1068.e1–3. 26384752 10.1016/j.jtcvs.2015.07.068

[30] Morbidity of bleeding after cardiac surgery: is it blood transfusion, reoperation for bleeding, or both? – PubMed. cited [2024 Sep 25]. https://pubmed.ncbi.nlm.nih.gov/21619974/10.1016/j.athoracsur.2011.03.105 21619974

[31] Vohra HA Ahmed EM Meyer A. Knowledge transfer and quality control in minimally invasive aortic valve replacement. Eur J Cardiothorac Surg 2018;53:ii9–13. 29718232 10.1093/ejcts/ezy077

[32] Minimally invasive versus conventional aortic valve replacement: The network meta - analysis Ogami 2022 - Journal of Cardiac Surgery - Wiley Online Library. [cited Sep 25 2024]. https://onlinelibrary.wiley.com/doi/10.1111/jocs.17126. 10.1111/jocs.17126 36378939

[33] Phan K Xie A Tsai YC. Ministernotomy or minithoracotomy for minimally invasive aortic valve replacement: a Bayesian network meta-analysis. Ann Cardiothorac Surg 2015;4:3–14. 25694971 10.3978/j.issn.2225-319X.2014.08.01 PMC4311162

[34] Evaluation of the incidence of new-onset atrial fibrillation after aortic valve replacement | valvular heart disease | JAMA internal medicine | JAMA Network. [cited 2024 Sep 25]. Available from: https://jamanetwork.com/journals/jamainternalmedicine/fullarticle/2734630 10.1001/jamainternmed.2019.0205 PMC6547161 31157821

[35] Brown ML McKellar SH Sundt TM. Ministernotomy versus conventional sternotomy for aortic valve replacement: a systematic review and meta-analysis. J Thorac Cardiovasc Surg 2009;137:670–679.e5. 19258087 10.1016/j.jtcvs.2008.08.010

[36] Messé SR Acker MA Kasner SE. Stroke after aortic valve surgery: results from a prospective cohort. Circulation 2014;129:2253–61. 24690611 10.1161/CIRCULATIONAHA.113.005084 PMC4043861

[37] Modi P Chitwood WR. Retrograde femoral arterial perfusion and stroke risk during minimally invasive mitral valve surgery: is there cause for concern? Ann Cardiothorac Surg 2013;2:E1. 24350000 10.3978/j.issn.2225-319X.2013.11.13 PMC3857048

[38] Boehme AK Esenwa C Elkind MSV. Stroke risk factors, genetics, and prevention. Circ Res 2017;120:472–95. 28154098 10.1161/CIRCRESAHA.116.308398 PMC5321635

[39] Claessens J Rottiers R Vandenbrande J. Quality of life in patients undergoing minimally invasive cardiac surgery: a systematic review. Indian J Thorac Cardiovasc Surg 2023;39:367–80. 37346428 10.1007/s12055-023-01501-y PMC10279589

[40] Reser D Walser R van Hemelrijk M. Long-term outcomes after minimally invasive aortic valve surgery through right anterior minithoracotomy. Thorac Cardiovasc Surg 2017;65:191–97. 27575273 10.1055/s-0036-1587591

[41] Merk DR Lehmann S Holzhey DM. Minimal invasive aortic valve replacement surgery is associated with improved survival: a propensity-matched comparison. Eur J Cardiothorac Surg 2015;47:11–7.discussion17. 24599160 10.1093/ejcts/ezu068

[42] Nkomo VT Gardin JM Skelton TN. Burden of valvular heart diseases: a population-based study. Lancet 2006;368:1005–11. 16980116 10.1016/S0140-6736(06)69208-8

[43] Baumgartner H Falk V Bax JJ. 2017 ESC/EACTS guidelines for the management of valvular heart disease. Eur Heart J 2017;38:2739–91. 28886619 10.1093/eurheartj/ehx391

